# SFI Enhances Therapeutic Efficiency of Gefitinib: An Insight into Reversal of Resistance to Targeted Therapy in Non-small Cell Lung Cancer Cells

**DOI:** 10.7150/jca.32989

**Published:** 2020-01-01

**Authors:** Zhenzhen Pan, Kai Wang, Qiufang Chen, Xiulan Zheng, Zhengyu Song, Xuansheng Ding

**Affiliations:** 1 China Pharmaceutical University, School of Basic Medicine and Clinical Pharmacy, Nanjing, 211198, China; 2 Xiamen Maternity and Child Health Care Hospital, Xiamen, 361000, China

**Keywords:** non-small cell lung cancer, EGFR-TKI, drug resistance, SREBP1, shenqi fuzheng injection

## Abstract

**Background:** The clinical application of EGFR tyrosine kinase inhibitors is always accompanied by inevitable drug resistance. However, the mechanism remains elusive. In the present study, we investigate the involvement of MAPK/SREBP1 pathway in NSCLC gefitinib resistance and evaluate the synergistic effects of shenqi fuzheng injection (SFI) and gefitinib on NSCLC cells.

**Methods:** To investigate the MAPK/SREBP1 pathway involved in gefitinib resistance, Western blotting was used to examine p-MEK, p-ERK and SREBP1 expression in PC-9 and PC-9/GR cells, MTT was used on cell proliferation, wound healing assay was used on cell migration. To detect the cooperative effects of SFI and gefitinib, clonogenic assay was used on cell proliferation. Apoptosis assay was analyzed by flow cytometry. Immunofluorescence was used to detect gefitinib binding to EGFR. Western blotting was used to detect whether SFI regulate the resistance to gefitinib via the suppression of MAPK/SREBP1 pathway.

**Results:** Our results showed that MAPK/SREBP1 pathway mediated resistance to gefitinib in NSCLC cells. MAPK pathway was found to directly target SREBP1 and inhibition of SREBP1 increased gefitinib sensitivity. In addition, SFI showed cooperative anti-proliferation and pro-apoptosis impacts on gefitinib resistant cells via down-regulating MAPK/SREBP1 pathway. Moreover, the combination of SFI and gefitinib enhanced gefitinib binding to EGFR resulting in the restoration of sensitivity to gefitinib.

**Conclusions:** Taken together, MAPK/SREBP1 pathway could be regarded as the potential treatment target for overcoming resistance to EGFR-TKIs in NSCLC and adjuvant therapy of SFI could be a potential therapeutic strategy for gefitinib resistant treatment.

## Introduction

Lung cancer is one of the most common types of cancer diagnosed worldwide and leads to high mortality 1, 2. Non-small cell lung cancer (NSCLC) accounts for 85% of all cases 3. Approximately 64% of patients with NSCLC harbor an oncogenic driver mutation, such as epidermal growth factor receptor (EGFR) and Kirsten rat sarcoma 2 viral oncogene homolog (KRAS), which leads to improvements in survival and safety compared with conventional chemotherapy 4. EGFR tyrosine kinase inhibitors (EGFR-TKIs) are effective in approximately 70% of NSCLC with EGFR activating mutation 5. Inevitably, the most of patients develop acquired resistance after 1 year treatment with EGFR-TKIs on average due to a variety of mechanisms 6.

The possible mechanisms of EGFR-TKIs resistance include second-site mutation of EGFR kinase domain, histological transformation, bypass signaling pathways activation including MAPK pathway and molecular changes to promote cell survival and inhibition of apoptosis 7. For instance, AZD9291 was discovered to overcome secondary resistance mutations, resistant cells can develop a bypass pathway to reactivate downstream proliferation and survival signal 8. It is necessary to figure out the complex and ambiguous resistant mechanisms and develop alternative therapeutic methods to overcome EGFR-TKIs resistance.

Our team has demonstrated that high intracellular level of cholesterol was the leading cause of gefitinib resistance in non-small cell lung cancer 9. Evidence showed that the expression of sterol regulatory element binding protein 1 (SREBP1) was high in tumor tissue 10.SREBP1 is a key transcription factor of lipid homeostasis and activates genes required for the synthesis of cholesterol 11. Inhibiting SREBP1 expression decreased the tumor growth *in vivo* 12. The signal transduction pathways, such as MAPK pathway, have been identified to regulate SREBP1 expression in cancer cells 13, 14. However, the role of MAPK/SREBP1 playing in EGFR mutation gefitinib resistant NSCLC cells has not been clarified.

Many Traditional Chinese medicine (TCM) have anticancer effects and can enhance the efficacy of EGFR-TKIs in NSCLC 15, 16. It is a promising strategy to combine TCM and EGFR-TKIs as anticancer treatment to overcome drug resistance. Shenqi fuzheng injection (SFI) is a modern TCM commonly used in clinic as an antitumor injection. Two Chinese medicine herbs, codonopsis and astragali are the main resources of SFI 17. It has been reported that combination of SFI and chemotherapy could improve the quality of life, reduce toxicity and exhibit synergistic antitumor effects in NSCLC patients 18, 19. However, the synergistic effects of SFI and gefitinib on gefitinib resistant NSCLC cells, which may become a promising strategy to overcome EGFR-TKIs resistance, and underlying mechanisms are poorly understood.

In the current study, the role of MAPK/SREBP1 pathway in NSCLC with resistance to gefitinib was assessed for the first time, and the potential therapeutic effect of targeting MAPK/SREBP1 pathway was examined in NSCLC cells.

## Materials and methods

### Chemicals and reagents

Shengqi fuzheng injection (Z19990065) was provided by livzon Pharmaceutics ltd. (Zhuhai, China). For cell culture, SFI was dissolved in DMEM to different concentration gradients. Gefitinib was purchased from Aladdin Industrial Corporation (Shanghai, China). The 3-(4,5-dimethylthiazol-2-yl)-2, 5-diphenyltetrazolium bromide (MTT) was purchased from Biosharp (Shanghai, China). Dulbecco's modified eagle medium (DMEM), DMSO, Penicillin- Streptomycin Solution, Annexin V-FITC kit were purchased from KeyGen (KeyGen, Nanjing, China). DAPI staining solution, EGF, BCA protein assay kit, Crystal Violet Staining Solution were purchased from Beyotime Biotechnology (Shanghai, China). Anti-p- EGFR(Tyr 1172), anti-EGFR, anti-MEK1/2, anti-p- ERK1/2(thr202/tyr204), anti-ERK1/2, anti-β-actin, goat anti-rabbit IgG H&L (HRP), anti-cleaved-caspase 3, anti-cleaved-caspase 9, anti-Bcl-2, anti-Bax antibodies were purchased from Wanlei Bio. (Shenyang, China). Anti-p-MEK1/2 (Ser217/221) antibody was purchased from Cell Signaling Technology (Cell Signaling Technology, Danvers, MA, USA).

### Cell culture

Human NSCLC H1650 and H1975 cells were obtained from 3D Medicines. Human NSCLC PC-9 and PC-9/GR cells were given by Dr. Zhou Caicun. Cells were cultured in DMEM containing 12% Fetal bovine serum (Biological Industries), Penicillin- Streptomycin Solution (1X) at 37℃ in an atmosphere of 5% CO_2_.

### Cell proliferation assay

The cell proliferation assay of gefitinib and SFI was measured by MTT. Cells in 96-well plates were treated with indicated drugs. Then, each well added 150 μL MTT solution incubated at 37℃ for 4 h. Absorbance was determined at 570 nm using a microplate reader (Thermo Multiskan_FC, American).

### Drug synergy analysis

The data from the PC-9/GR, H1975 and H1650 cell proliferation assays was analyzed by CompuSyn software (Biosoft, Cambridge, UK) to investigate the synergistic effects of SFI and gefitinib on cells *in vitro*. The combination index (CI)-isobologram equation was described as previously 20.

### Clonogenic assay

500 cells per well were seeded into 12-well plates and cultured in DMEM supplemented with no drug solution, SFI (1:10), Gefitinib 4 μM or in combination at 37˚C_._ After 14 days, the cells were fixed with 4% paraformaldehyde and stained with crystal violet solution. Finally, the plates were washed dried at room temperature and photographed.

### Wound healing assay

To evaluate the migration ability of cells, a wound healing assay was performed. The cells were seeded in 96-well plates and then, as its growth to 80% confluence, the wounds were scratched with pipette tip across the center of the well. After having been washed, the cells were incubated in humid atmosphere. The migrated cells were observed via the optical microscope at 0 and 12 h, respectively.

### Apoptosis assay

To test apoptosis, cells were planted in 6-well plates incubated with indicated drugs for 24 h before cell dissociation and assemblage. The cells were harvested and re-suspended in 500 µL binding buffer. The cells were then stained with 5 µL Annexin V-FITC and Propidium Iodide for 5-10 min in the dark condition. Flow cytometry analysis (Becton Dickinson FACS Calibur; Becton-Dickinson, USA) was conducted to detect apoptosis.

### Western blotting assay

Cells were treated with indicated drugs. Then the total protein was collected from cells. The concentration of protein in the supernatants was detected according to BCA protein assay kit instructions (Beyotime, P0010). Then, 60 μg protein was separated by 8%-12% SDS-PAGE and analyzed with antibodies. The final detection was performed by ECL reagents (KeyGen, KGP1121).

### Immunofluorescence assay

Cells (1×10^5^ cells/mL) were cultured on dish and treated with fluorescent-labeled quinazoline skeleton of gefitinib (10 µM) alone or in combination with SFI (1:10) for 3 h. Cells were washed with PBS, then incubated within DAPI (Beyotime, C1006) for further 20 min. Finally, the images were gained by a ZEISS.

## Results

### Gefitinib induced cytotoxicity in NSCLC cells with high constitutive levels of MAPK and SREBP1

**Figure 1:**
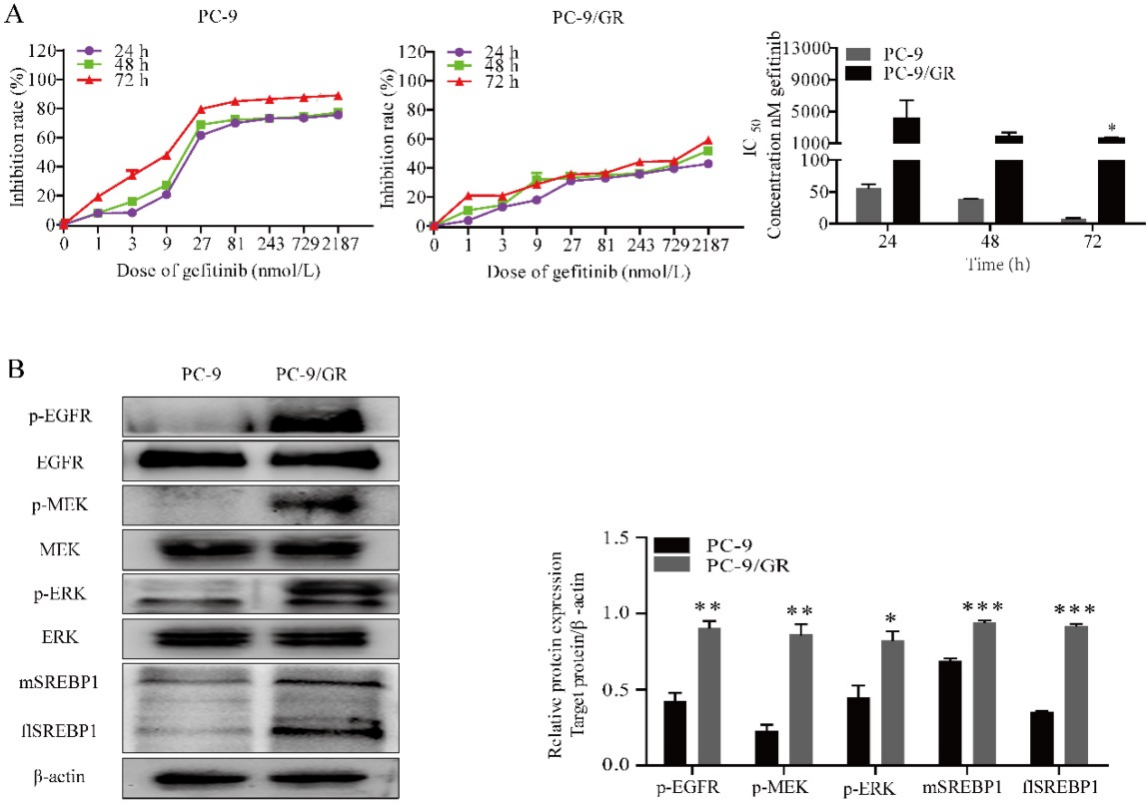
** Gefitinib suppresses PC-9 and PC-9/GR cell proliferation. (A)** Growth inhibition assay of gefitinib was determined by MTT under indicated concentration of gefitinib. **(B)** Western blotting for p-EGFR, EGFR, p-MEK, MEK, p-ERK, ERK, flSREBP1 and mSREBP1 protein expression in PC-9 and PC-9/GR cells treated with EGF. *p<0.05, **p<0.01 or ***p<0.001 compared to PC-9 cells.

We chose gefitinib sensitive PC-9 cells harboring EGFR exon 19 deletion and gefitinib resistant PC-9/GR cells for experiment. To quantify gefitinib cytotoxicity, MTT assay was performed. The cells were incubated for 24, 48 or 72 h at indicated doses of gefitinib (1, 3, 9, 27, 81, 243, 729 and 2187 nmol/L). As shown in Fig. 1A, the results indicated that gefitinib significantly inhibited the proliferation of PC-9 cells in a time and dose-dependent manner, and slightly inhibited the proliferation of PC-9/GR cells.

To test whether MAPK signaling cascades phosphorylated and activated in cells with resistance to gefitinib, western blotting was conducted. As expected, phosphorylated MEK and ERK were elevated in PC-9/GR compared with PC-9 cells (Fig. 1B). Since it has been reported that MAPK pathway affected the transcriptional activity of SREBP1, directly 21. Here, we compared the expression of SREBP1 in different cells. Notably, we found a higher expression of both flSREBP1and mSREBP1 in PC-9/GR cells.

### Inhibition of SREBP1 reversed Gefitinib resistance of NSCLC cells

**Figure 2:**
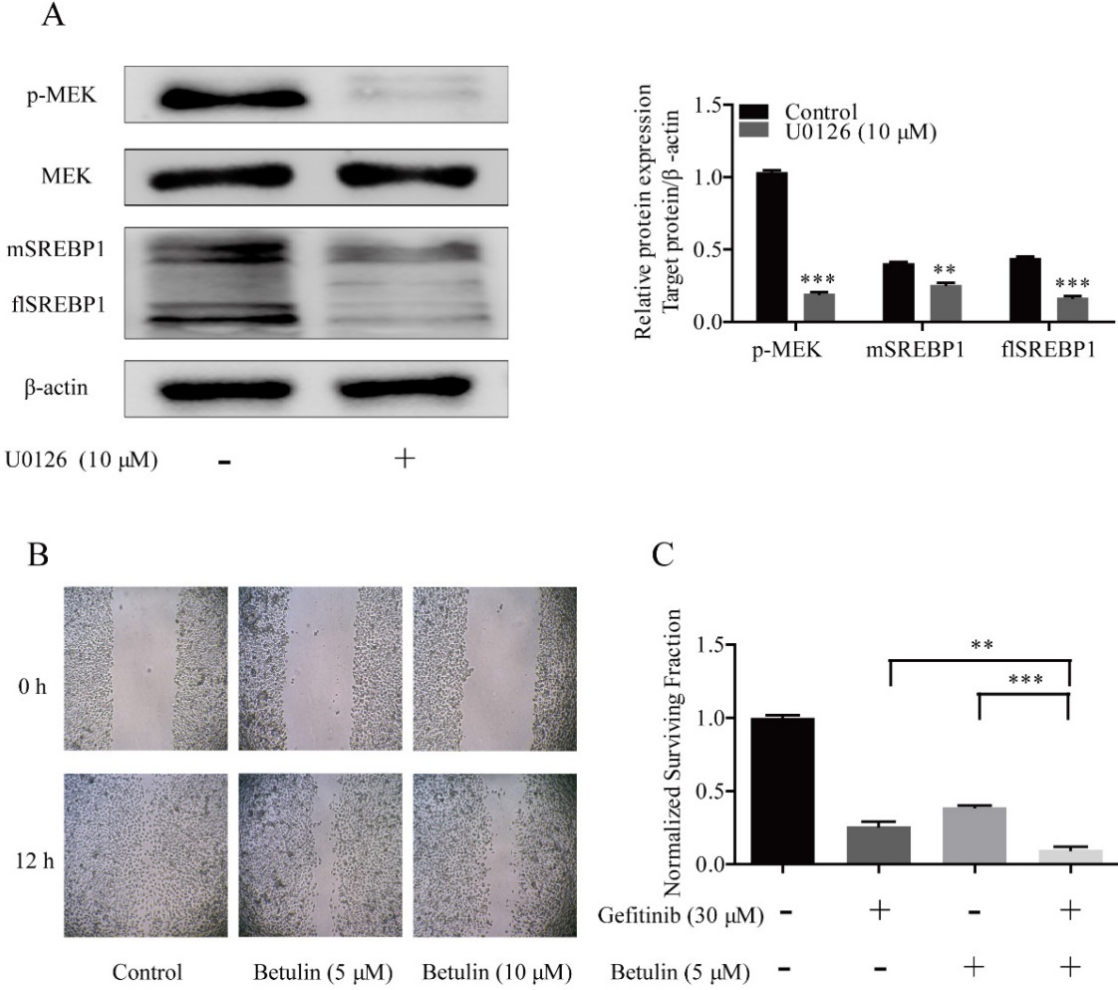
** Effects of MPAK pathway inhibition on SREBP1 expression and SREBP1 inhibition on migration and proliferation in PC-9/GR cells. (A)** Western blotting for p-MEK, MEK, flSREBP1 and mSREBP1 protein expression in PC-9/GR cells after being treated with 10 μM U0126. **p<0.01 or ***p<0.001 compared to control group. **(B)** Cells were treated with gradient concentration of betulin (5 and 10 μM). Closed wound area of 5 and 10 μM betulin group at 12 h were significantly smaller compared to Control group. **(C)** PC-9/GR cells were treated with betulin 5 μM alone or combined with gefitinib 30 μM for 48 h. Relative cell viability was measured by MTT assay. **p<0.01 or ***p<0.001 compared to combination group.

U0126 is a selective inhibitor of MEK kinases 22, which has been widely used as an inhibitor for the MAPK pathway in diverse fields 23. To confirm the effects of MAPK pathway on SREBP1 expression, PC-9/GR cells were treated with 10 μM U0126. We found that both flSREBP1 and mSREBP1 expression were inhibited by U0126.These data indicated that overexpression of SREBP1 was due to activated MAPK pathway in PC-9/GR cells (Fig. 2A).

Betulin was previously identified as an inhibitor of SREBP pathway 24. Treating cells with gradient concentration of betulin, the migration of PC-9/GR cells was inhibited (Fig. 2B). To examine whether inhibit SREBP1 could reverse cells resistance to gefitinib, 5 μM betulin was then combined with 30 μM gefitinib. The combined treatment exhibited a greater anti-proliferation effect on PC-9/GR cells than a single drug alone (Fig. 2C). These data confirmed that the MAPK/SREBP1 pathway mediated resistance to gefitinib in NSCLC cells.

### SFI synergizes with gefitinib to inhibit cell proliferation and clonogenicity in PC-9/GR, H1975 and H1650 cells

**Figure 3:**
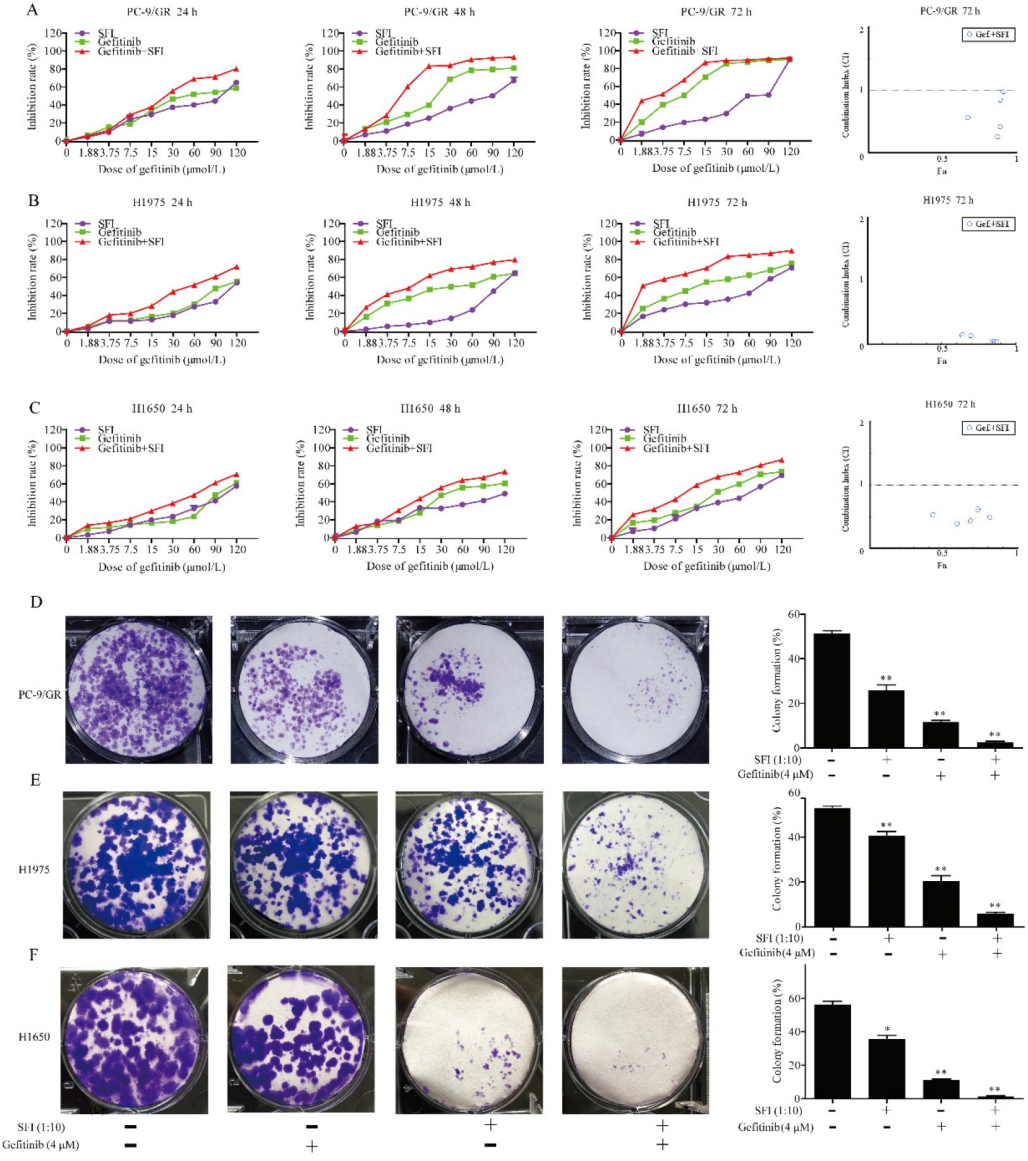
** The synergistic effects of SFI and gefitinib on cell proliferation and clonogenicity. (A, B and C)** Cells were treated with gefitinib alone or in combination with SFI for 24, 48 or 72 h. MTT was used to detect the cell viability. The Fa-CI (Combination Index) Plot was generated by CompuSyn software. Points that fall below the line indicate synergic relationship between the two drugs. The CI was calculated using the data and CompuSyn software. **(D, E and F)** Clonogenic assays were performed in PC-9/GR, H1975 and H1650 cells with no drug solution, SFI (1:10), Gefitinib 4 μM alone or in combination. *p<0.05 or **p<0.01 compared to control group.

Shenqi fuzheng injection (SFI) was extracted from astragali and codonopsis, which was generally used to improve the immune system of patients with NSCLC 25. Previous study has reported that astragaloside IV inhibited accumulation and nuclear translocation of SREBP1 26. We presumed that SFI might show synergistic antitumor effects with gefitinib by inhibiting SREBP1 expression in NSCLC cells. To investigate the synergistic effects on NSCLC cell motility, PC-9/GR, H1975 and H1650 cells were selected for further study. Cells were treated with different concentrations of gefitinib (1.875, 3.75, 7.5, 15, 30, 60, 90 and 120 μmol/L), SFI (1:1, 1:2, 1:4, 1:8, 1:16, 1:32, 1:64, 1:128) alone or in combination for 24, 48 or 72 h. The combined treatment had significant inhibition on PC-9/GR, H1975 and H1650 cells when compared with a single drug alone. (Fig. 3A, B and C). The results of the CI-isobologram analysis showed that SFI and gefitinib had synergistic effects on the PC-9/GR (CI: 0.179-0.982), H1975 (CI: 0.032-0.582), and H1650 (CI: 0.360-0.834) cells at 72 h. The combination of SFI and gefitinib rendered the PC-9/GR, H1975 and H1650 cells more sensitive to gefitinib. To further evaluate the synergistic anti-cancer effects of gefitinib and SFI, clonogenic assay was performed. We found that SFI, gefitinib alone or in combination inhibited cell colony formation in all three kinds of cells. Furthermore, combined treatment could significantly reduce the number of clones compared to each drug alone (Fig. 3D, E and F).

### SFI enhances the effect of gefitinib on inducing apoptosis in NSCLC cells

**Figure 4:**
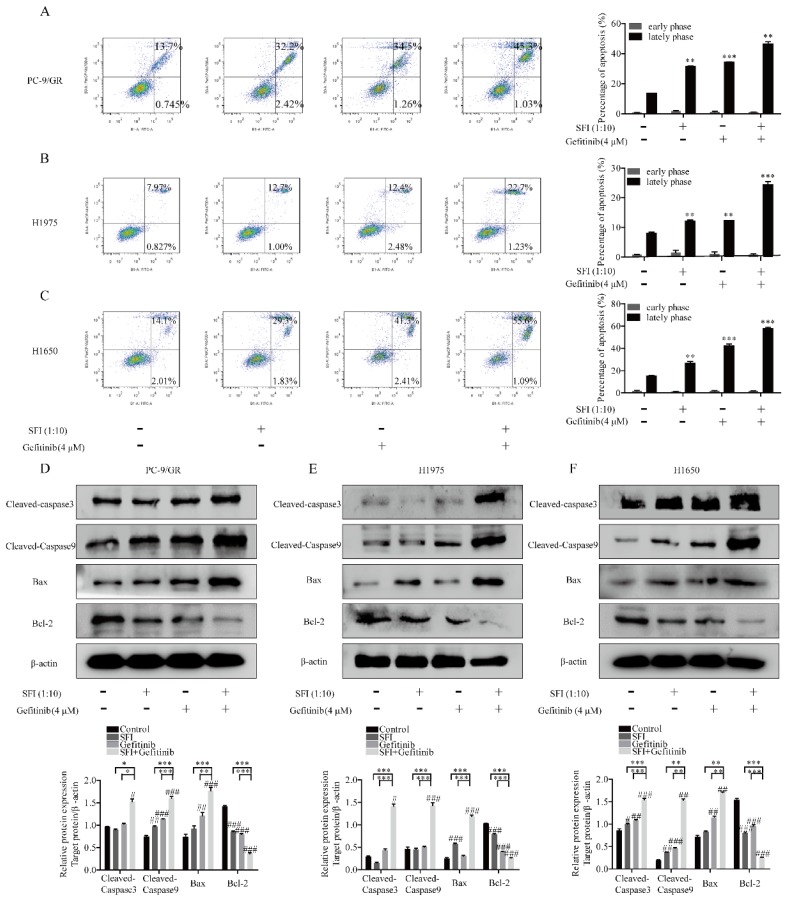
** SFI synergizes with Gefitinib to induce apoptosis. (A, B and C)** Apoptosis assay was conducted with SFI (1:10) and Gefitinib 4 μM alone or in combination for 24 h by Flow cytometry. **p<0.01 or ***p<0.001 compared to control group. **(D, E and F)** Western blotting was used to detect cleaved-caspase 3, cleaved-caspase 9, bax, and Bcl-2 protein expression after being treated with SFI (1:10) and Gefitinib 4 μM alone or in combination for 24 h. *#p*<0.05, *##p*<0.01 or *###p*<0.001 compared to control group. *p<0.05, **p<0.01 or ***p<0.001 compared to combination group.

To test the regulation of SFI on cell apoptosis induced by gefitinib, cells were co-treated with SFI and gefitinib. As shown in Fig. 4A-C, SFI synergized with gefitinib in inducing cell apoptosis. The combined treatment increased cleaved-caspase 3, cleaved-caspase 9 and pro-apoptotic proteins Bax expression, as well as decreased the expression of anti-apoptotic protein Bcl-2. (Fig. 4D, E and F).

### The synergistic efficacy of SFI and gefitinib is dependent on inhibition of MAPK/SREBP1 pathway

**Figure 5:**
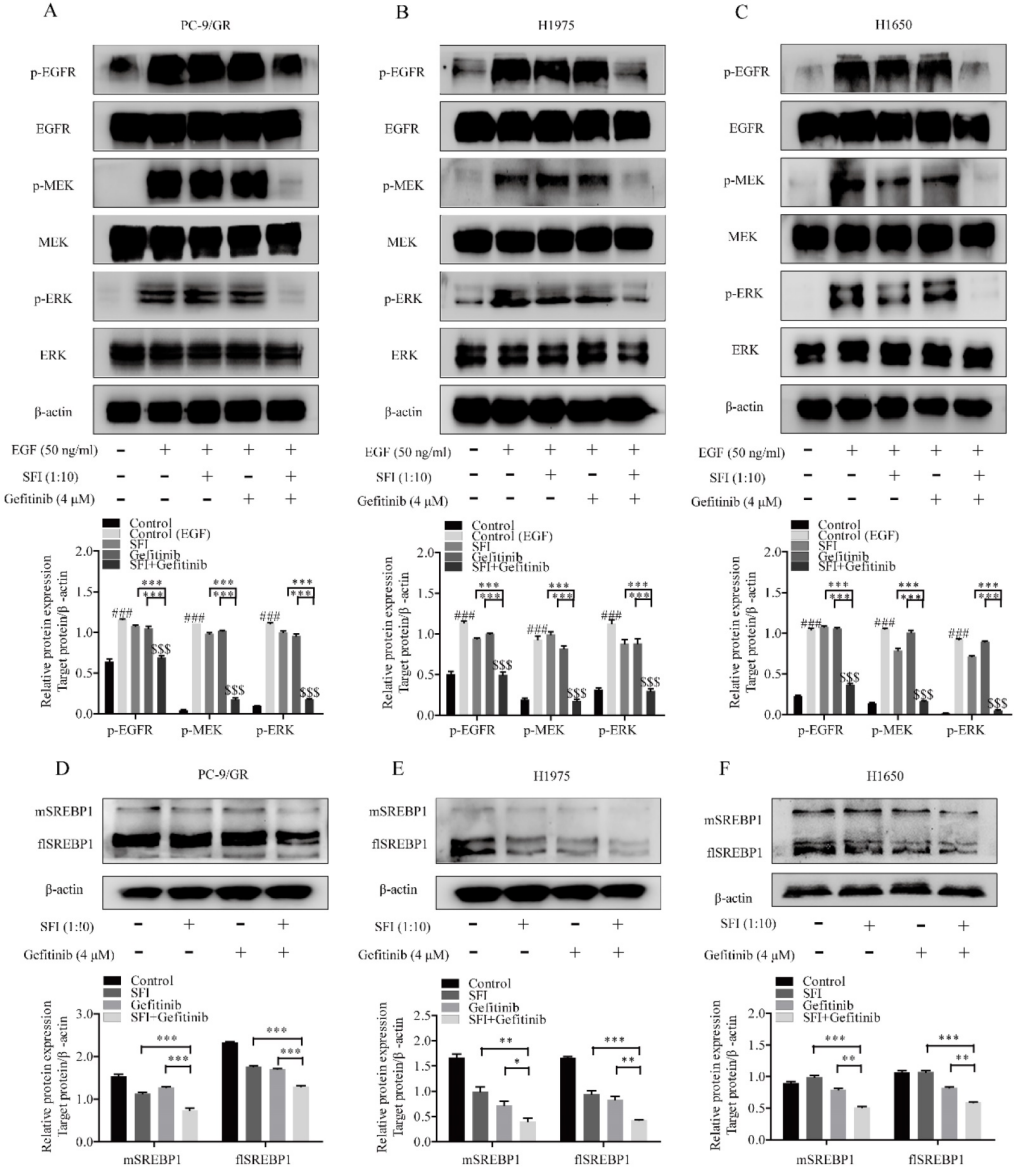
** The mechanism of SFI showing synergistic efficacy on cells with Gefitinib is inhibiting MAPK/SREBP1 pathway. (A, B and C)** Western blotting was performed to determined p-EGFR, EGFR, p-MEK, MEK, p-ERK, ERK protein expression after being treated with SFI (1:10) and Gefitinib 4 μM alone or in combination in PC-9/GR, H1975 and H1650 cells. *###p*<0.001 compared to control group.* $$$p*<0.001 compared to control (EGF) group. ***p<0.001 compared to combination group. **(D, E and F)** Western blotting was conducted to detect flSREBP1 and mSREBP1 protein expression after being treated with SFI (1:10) and Gefitinib 4 μM alone or in combination in PC-9/GR, H1975 and H1650 cells. *p<0.05, **p<0.01 or ***p<0.001 compared to combination group.

To elucidate whether a possible mechanism of synergy is due to the involvement of regulating MAPK/SREBP1 pathway, cells were treated with SFI and gefitinib respectively, or in combination for 24h, and then analyzed EGFR-related proteins levels by western blotting. As shown in Fig. 5A-C, gefitinib and SFI alone had no regulatory effects on phosphorylated EGFR, MEK, ERK proteins levels. Nevertheless, combined treatment showed a significantly inhibition on p-EGFR, p-MEK and p-ERK expression. We then investigated the expression of flSREBP1 and mSREBP1, a similar tendency was observed (Fig. 5D, E and F). To sum up, the combination of SFI and gefitinib could be a potential therapeutic strategy for gefitinib resistant treatment in NSCLC cells via regulating MAPK/SREBP1 pathway.

### SFI enhances gefitinib binding to EGFR resulting in restoration of sensitivity to gefitinib in PC-9/GR and H1975 cells

**Figure 6:**
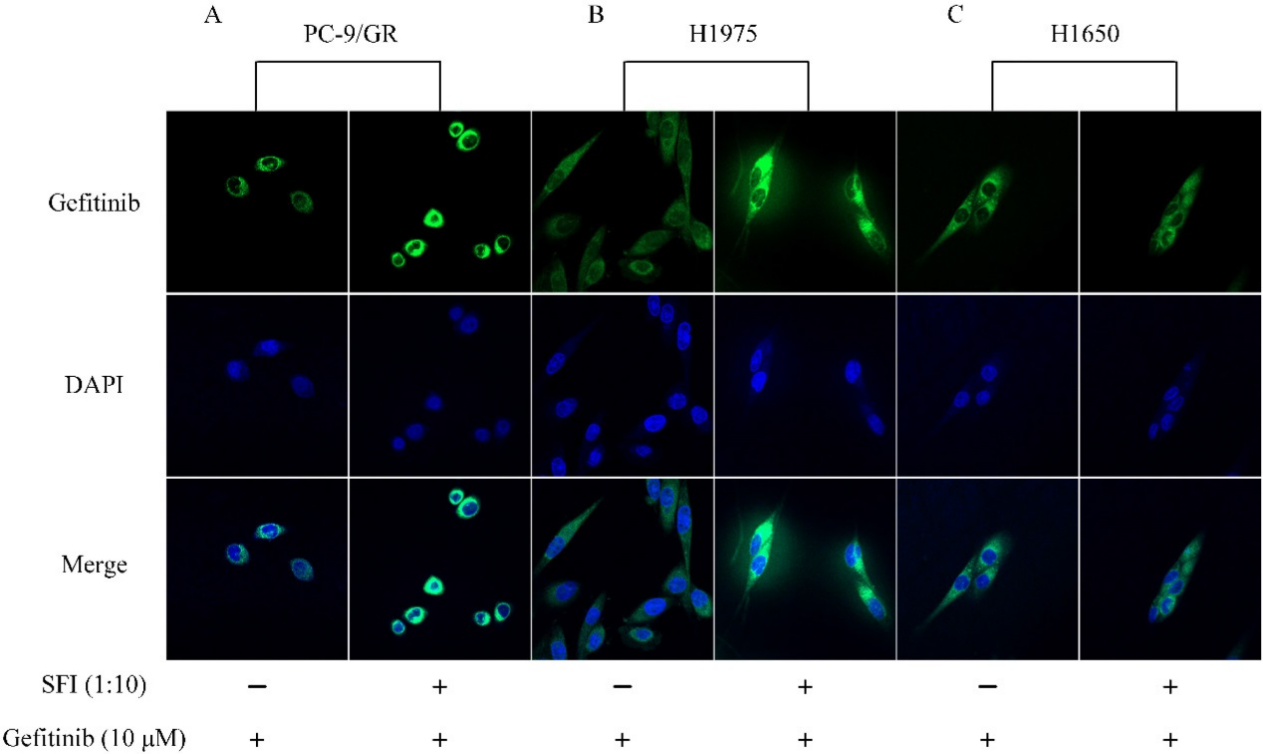
** SFI enhances Gefitinib binding to EGFR in PC-9/GR, H1975 cells. (A, B and C)** Cells were exposed to fluorescent labeled gefitinib quinazoline skeleton (10 µM) alone or in combination with SFI (1:10) for 3 h. Immunofluorescence assay was conducted to detect the affinity of gefitinib to EGFR tyrosine kinase domain (green fluorescence).

SREBP1 is a transcription factor that maintain cellular lipid homeostasis by regulating the expression of many enzymes needed for the formation of cholesterol and fatty acid. Cholesterol and fatty acid are main components of mammalian cell membrane. EGFR is known to be a plasma membrane-resident protein, whose function is modulated by its surrounding lipid environment 27. To determine whether SFI can cause changing in gefitinib affinity to EGFR, cells were treated with gefitinib alone or in combination with SFI. The fluorescence intensity was represented for the binding capacity of gefitinib to EGFR. Enhanced fluorescence intensity was observed by Confocal imaging (Fig. 6A, B and C) when cells were co-treated with SFI and gefitinib in PC-9/GR and H1975 cells. These results revealed that SFI increased gefitinib affinity in acquired resistant PC-9/GR and H1975 cells, but not in primary resistant H1650 cells.

## Discussion

Gefitinib is the first EGFR-TKI that was approved for the therapy of patients with NSCLC 28. By competitively interacting with the ATP-binding site, gefitinib can inhibit EGFR kinase activity, prevent auto-phosphorylation and suppress downstream signaling. NSCLC patients harboring EGFR mutation demonstrate good responses to gefitinib. Unfortunately, the clinical application of gefitinib is limited by drug resistance due to many mechanisms including the secondary T790M mutation, a most common mechanism for gefitinib resistance manifested in approximately 60% of patients. The third generation EGFR-TKIs, such as osimertinib, is designed to overcome T790M mutation. This new agent significantly increases the overall response rates of patients. However, similar to gefitinib, the application of osimertinib has been accompanied by the drug resistance. Several mechanisms of resistance have been identified including EGFR C797S mutation, MET amplification and epithelial-mesenchymal transition (EMT) 29. Even with the fourth generation EGFR-TKIs on the clinical research, the complex mechanisms of drug resistance have not been fully revealed. Thus, there is a need to understand the underlying mechanism and identify the key molecule target so as to develop new strategies to overcome EGFR-TKIs resistance.

The study is based on our previous work which showed that high levels of cholesterol in lipid rafts are responsible for gefitinib resistance in NSCLC cells and the depletion of cholesterol can restore the sensitivity of gefitinib. We presumed that the key molecules involved in the regulation of cellular cholesterol level could be targets to overcome EGFR-TKIs resistance. SREBP1 is a key transcription factor for cholesterol homeostasis by regulating the transcriptional activation of target genes, such as 3-hydroxy-3-methylglutaryl-CoA reductase (HMGCR) and low-density lipoprotein receptor (LDLR) 30. In the present study, we found a higher expression of SREBP1 in PC-9/GR cells compared to PC-9 cells (*p*<0.001). As it was documented before, SREBP1 could promote proliferation, metastasis and EMT in cancer cells by providing the membrane building materials 31. We acquired similar results where the suppression of SREBP1 by betulin inhibited the migration of PC-9/GR cells. Further study was conducted to investigate the role of SREBP1 playing in gefitinib resistance by combining betulin and gefitinib to treat cells. Results showed that inhibition of SREBP1 enhanced cell sensitivity to gefitinib in NSCLC cells.

The Ras-Raf-MEK-ERK mitogen-activated protein kinase (MAPK) pathway governs fundamental physiological processes, such as cell proliferation, metabolism, cell death and survival in NSCLC 32. It is activated by extracellular ligands, such as epidermal growth factor (EGF), and motivates cell survival by regulating a range of targets including caspase 3, caspase 9, Bcl-xl and Bad transcription factors 33. We found MAPK signaling cascades phosphorylated and activated in PC-9/GR cells. Previous studies have identified SREBP as a downstream effector of MAPK cascades in prostate cancer or melanoma, but not in NSCLC. To determine the targeted relationship between MAPK pathway and SREBP1 in NSCLC gefitinib resistant cells, we pharmacologically inhibited MAPK pathway by U0126. We discovered that in PC-9/GR cells, the expression of SREBP1 (both flSREBP1 and mSREBP1) was regulated by MAPK pathway. Our results demonstrated that MAPK/SREBP1 pathway was responsible for gefitinib resistance in NSCLC cells.

Regarding SFI are mainly composed of codonopsis and astragali. Previous studies have reported that inhibitory impacts of astragaloside on cancer cells were probably related to its regulating MAPK pathway 34, 35. Moreover, astragaloside IV inhibited the accumulation and nuclear translocation of mature SREBP1 36. Above all, we presumed that SFI might show synergistic antitumor effects of gefitinib by regulating MAPK/SREBP1 pathway in NSCLC gefitinib resistant cells. Here, we selected PC-9/GR, H1975 and H1650 cells to detect synergistic effects of SFI and gefitinib. Results suggested that SFI combined with gefitinib to inhibit cell proliferation, clonogenicity and induce apoptosis, which were consistant with the study conducted by Xiong Y, et al. They documented that SFI increased chemotherapy sensitivity in cisplatin resistance of NSCLC cells through regulating cell cycle and initiating mitochondrial apoptosis 37. We further validated the underlying mechanism of SFI reversing gefitinib resistance was the inhibition of MAPK/SREBP1 pathway.

The secondary T790M mutation within the ATP site of EGFR is the most common mechanism of resistance to the first generation EGFR-TKIs in lung cancers 38, which reduces the binding efficacy of EGFR-TKIs to EGFR kinase domain 39. The EGFR is a membrane-bound receptor, which consists of an extracellular module and an intracellular kinase domain. The activation and function of EGFR is related to membrane lipids 40. Main components of membrane such as phospholipids, fatty acids and cholesterol have been reported to regulate drug sensitivity of gefitinib in NSCLC 41, 42. SREBP1 is the key factor transcription factor playing a central role in lipid metabolism. Based on the result that SFI combined with gefitinib can reduce the SREBP1 protein expression. Here, we speculated that SFI might enhance gefitinib binding to EGFR by inhibiting SREBP1 and then detected the binding of gefitinib to EGFR by Immunofluorescence. We found that SFI enhanced gefitinib binding to EGFR in acquired resistant NSCLC PC-9/GR and H1975 cells, but not in primary resistant H1650 cells. The resistant mechanism of H1650 cells is associated with PTEN deletion without affecting gefitinib binding to EGFR 43. Therefore, it is not surprising that SFI did not exert any impacts on binging gefitinib to EGFR in H1650 cells.

In summary, our data initially exhibit the MAPK/SREBP1 pathway were responsible for gefitinib resistance in NSCLC cells and draw a conclusion that the combined treatment of SFI augmented gefitinib's anti-proliferation and pro-apoptosis potential in NSCLC gefitinib resistant cells through regulating MAPK/SREBP1 pathway. Moreover, the combined treatment enhanced gefitinib binding to EGFR resulted in restoration of sensitivity to gefitinib in acquired resistant NSCLC cells. Thus it can be seen that inhibition of MAPK/SREBP1 pathway is a prospective strategy to conquer gefitinib resistance in NSCLC cells and adjuvant therapy of SFI could be a potential therapeutic strategy for gefitinib resistant treatment.

## Abbreviations

AKT: protein kinase B
ALK: anaplastic lymphoma kinase
ATP: adenosine triphosphate
BCA: bicinchoninic acid
CI: combination index
DAPI: 4′:6-diamidino-2-phenylindole
DMEM: dulbecco's modified eagle medium
DMSO: dimethyl sulfoxide
EGF: epidermal growth factor
EGFR: epidermal growth factor receptor
EGFR-TKIs: EGFR tyrosine kinase inhibitors
EMT: epithelial-mesenchymal transition
flSREBP1: full length sterol regulatory element binding protein 1
HMGCR: 3-hydroxy-3-methylglutaryl-CoA reductase
KRAS: kirsten rat sarcoma 2 viral oncogene homolog
LDLR: low-density lipoprotein receptor
MAPK: mitogen-activated protein kinase
MET: mesenchymal-epithelial transition
mSREBP1: mature sterol regulatory element binding protein 1
MTT: 3-(4:5-dimethylthiazol-2-yl)-2:5-diphenyltetrazolium bromide
NSCLC: non-small cell lung cancer
PBS: phosphate buffer solution
PI3K: phosphatidylinositol-4:5-bisphosphate 3-kinase
PTEN: phosphatase and tensin homolog
SFI: shenqi fuzheng injection
SREBP1: sterol regulatory element binding protein 1
TCM: traditional Chinese medicine
